# Transmural Intestinal Wall Permeability in Severe Ischemia after Enteral Protease Inhibition

**DOI:** 10.1371/journal.pone.0096655

**Published:** 2014-05-07

**Authors:** Angelina E. Altshuler, Itze Lamadrid, Diana Li, Stephanie R. Ma, Leena Kurre, Geert W. Schmid-Schönbein, Alexander H. Penn

**Affiliations:** Department of Bioengineering, The Institute of Engineering in Medicine, University Of California San Diego, La Jolla, California, United States of America; University of California, Los Angeles, United States of America

## Abstract

In intestinal ischemia, inflammatory mediators in the small intestine's lumen such as food byproducts, bacteria, and digestive enzymes leak into the peritoneal space, lymph, and circulation, but the mechanisms by which the intestinal wall permeability initially increases are not well defined. We hypothesize that wall protease activity (independent of luminal proteases) and apoptosis contribute to the increased transmural permeability of the intestine's wall in an acutely ischemic small intestine. To model intestinal ischemia, the proximal jejunum to the distal ileum in the rat was excised, the lumen was rapidly flushed with saline to remove luminal contents, sectioned into equal length segments, and filled with a tracer (fluorescein) in saline, glucose, or protease inhibitors. The transmural fluorescein transport was determined over 2 hours. Villi structure and epithelial junctional proteins were analyzed. After ischemia, there was increased transmural permeability, loss of villi structure, and destruction of epithelial proteins. Supplementation with luminal glucose preserved the epithelium and significantly attenuated permeability and villi damage. Matrix metalloproteinase (MMP) inhibitors (doxycycline, GM 6001), and serine protease inhibitor (tranexamic acid) in the lumen, significantly reduced the fluorescein transport compared to saline for 90 min of ischemia. Based on these results, we tested in an in-vivo model of hemorrhagic shock (90 min 30 mmHg, 3 hours observation) for intestinal lesion formation. Single enteral interventions (saline, glucose, tranexamic acid) did not prevent intestinal lesions, while the combination of enteral glucose and tranexamic acid prevented lesion formation after hemorrhagic shock. The results suggest that apoptotic and protease mediated breakdown cause increased permeability and damage to the intestinal wall. Metabolic support in the lumen of an ischemic intestine with glucose reduces the transport from the lumen across the wall and enteral proteolytic inhibition attenuates tissue breakdown. These combined interventions ameliorate lesion formation in the small intestine after hemorrhagic shock.

## Introduction

Intestinal ischemia is an important problem in critical care that can be caused by trauma or sepsis and is accompanied by an increase in small intestine permeability as measured by transport from the intestinal lumen into the blood [1]–[4]. The reduced perfusion to the intestine results in damage to the intestinal villi and other components of the intestinal wall [5], [6]. The permeability increases and, as a result, intestinal contents may leak across the mucosal barrier [7], [8]. After escape from the intestinal lumen, intestinal contents can be transported through the venous intestinal vasculature [9], [10], lymphatics [11], [12], or via the peritoneum into the systemic circulation [13], [14], and may be responsible for distant organ injury [12], [15]. While many studies have investigated the transport of material into the blood and lymphatics from the intestine, few have investigated the importance of the transmural permeability in mammalian species, a route that provides direct access to peripheral organs, despite its association with poor outcome and death [11], [14], [16]. Furthermore, few models have elucidated the effects of the luminal contents on deterioration of the intestine during ischemia.

In a severe ischemic state, there may be multiple mechanisms for breakdown of the intestine, e.g. by depletion of ATP, including cell apoptotic processes [5], and proteolytic degradation. We have shown previously that enteral treatment with protease inhibitors is protective during shock [10], [17]–[19], but since low molecular weight inhibitors such as tranexamic acid may also be transported into the wall of the intestine, determining their mechanism of action is confounded by the presence of both pancreatic-derived digestive proteases in the intestinal lumen and proteases inherent to the intestinal tissue, and even bacterial proteases [20], [21].

Several potential sources of proteases in the intestinal tissue could be activated during ischemia and may contribute to the breakdown of the intestinal wall. One of the most prevalent classes of protease in the epithelial cells and the wall of the intestine are the matrix metalloproteinases (MMPs), capable of digesting the extracellular matrix [22], [23]. Endothelial cells in microvessels, and extravasated leukocytes are also potential sources of MMPs [24], [25]. If activated or released during ischemia, these enzymes could degrade the intestinal wall, enabling leakage of pro-inflammatory mediators derived from the lumen (proteases, bacteria, digested food particles) of the intestine into the peritoneum [13], [14], [26]–[29].

The objective of this study is to investigate the breakdown of the wall of the small intestine during ischemia by mechanisms inherent to the tissue, i.e. in the absence of luminal contents, and determine which degrading processes (cell death or protease degradation) contribute to transmural permeability of a low molecular weight tracer. We hypothesize that in a model of severe intestinal ischemia metabolic support (e.g. glucose, which can be directly metabolized by enterocytes to ATP and has reduced epithelial shedding into the lumen during intestinal ischemia [30]–[32], [32,33]) helps to *preserve the epithelial barrier* resulting in minimal penetration of a low molecular weight tracer while enterally applied protease inhibitors may prevent *intestinal wall tissue breakdown*.

To test this hypothesis, we used an ex-vivo approach [26], in which luminal content of an excised intestine is replaced with a low molecular weight tracer in saline with glucose as metabolic support and/or protease inhibitors that were recently tested as enteral interventions in shock models. Transmural permeability, morphological damage, the level of protease activity in the tissue, and junctional protein integrity were determined. We chose this approach over in vivo models of total ischemia such as splanchnic arterial occlusion and ligation of vessels along isolated intestinal loops for two reasons: 1) complete ischemia is not assured by splanchnic arterial occlusion and 2) because of anatomical constraints, neither in vivo approach allows permeability to be measured as a function of distance along the intestine in the same animal. We report for the first time the change in transmural permeability after total ischemia along the entire length of the jejunum and ileum. Our results indicate that metabolic support to the epithelium preserves the mucosal barrier while enteral protease inhibition using tranexamic acid, a compound typically administered as an inhibitor of trypsin-like proteases but which we show also inhibits intestinal MMPs, prevents structural breakdown of the wall. We then show that in a model of hemorrhagic shock, treatment with glucose or tranexamic alone is not sufficient to prevent intestinal hemorrhage or a decline in blood pressure after reperfusion. However, enteral tranexamic acid plus glucose is effective at reducing transmural permeability and intestinal breakdown ex-vivo, preventing visible hemorrhage following hemorrhagic shock, and stabilizing blood pressure during reperfusion. Understanding the breakdown process of the intestinal wall may help in the design and implementation of new interventions against the escape of luminal contents into the peritoneum in conditions of intestinal ischemia.

## Methods

### Animal Models

The animal protocol was reviewed and approved by the University of California, San Diego Institutional Animal Care and Use Committee (Protocol Number S01113). Male Wistar rats (body weight between 250–400 g, Harlan, Indianapolis, IN) for the intestinal ischemia model and male Sprague Dawley rats (body weight between 255–435 g, Harlan) for the hemorrhagic shock model were allowed food and water *ad libitum* prior to surgery. All rats were administered general anesthesia (xylazine, 4 mg/kg; ketamine 75 mg/kg IM.) and euthanized with B-Euthanasia (120 mg/kg).

#### Intestinal Ischemia

Since intestinal properties are non-homogenous [34], [35], the transmural permeability was investigated over the entire length of the jejunum and ileum. Due to physical constraints of the small intestine anatomy, it is not feasible to simultaneously analyze permeability from multiple segments in-vivo. Therefore, an ex-vivo approach similar to previously published studies [26], [36] was designed to measure permeability along the length of the small intestine.

A midline incision was made to expose the intestine in anesthetized rats (4 mg/kg xylazine and 75 mg/kg ketamine i.m.). The proximal end of the jejunum (approximately 5 cm distal from the ligament of Treitz) was cannulated with a female luer to barb tube connector (1/8” inner diameter). The intestine was removed and placed in saline immediately before euthanasia. In order to focus the analysis on permeability of the intestinal wall during ischemia in the absence of luminal contents (including the digestive proteases or cytotoxic factors from food), the lumen of the intestine was flushed with 40 ml saline using pulsatile pressure. Visual examination confirmed all solid contents were removed. Next, the intestine was cut into eight equal length segments (∼8 cm) sequentially ordered from the proximal jejunum (position 1) to the distal ileum (position 8). Both ends of each segment were cannulated with Female Luer to Barb tube connectors (thus allowing entry and exit points from the intestinal lumen to be sealed completely), secured with 4-0 suture, and the exterior portion of one adaptor on each segment sealed with clay.

To determine the transmural permeability of the intestine, all 8 segments from each animal were filled with a low molecular weight tracer as a sensitive measure of early barrier breakdown prior to the initiation of ischemia, fluorescein (332 Da M.W., 20 µg/ml from 5 mg/ml stock in ethanol; 0.308 osm/L; Sigma-Aldrich, St. Louis, MO) mixed with saline, glucose (100 mg/ml in saline; 0.550 moles/L; 0.863 osm/L, Sigma-Aldrich), the non-metabolizing glucose analog mannitol (100 mg/ml in saline; 0.550 moles/L; 0.863 osm/L), the serine protease and MMP inhibitor tranexamic acid [6], [17], [18] (31 mg/ml in saline; 0.200 moles/L; 0.508 osm/L, Sigma-Aldrich), the MMP inhibitor doxycycline hyclate [37] (1 mg/ml in saline; 1.955×10^−3^ moles/L; 0.310 osm/L, Sigma-Aldrich), or the MMP inhibitor GM 6001 [38] (1 µg/ml in saline from 1 mg/ml stock in DMSO; 2.57×10^−6^ moles/L; 0.308 osm/L, Millipore, Billerica, MD). Concentrations were chosen to match those previously used in in vivo shock studies (e.g. the tranexamic acid concentration is in the range that was previously used to inhibit enteral trypsin) [6], [17], [18].

300 µl of sample were added to each intestinal segment (this volume does not fully inflate or stretch the intestinal tissue) through the tubing adaptor before sealing with clay. Sealed segments were rinsed in saline, individually placed in 15 ml conical tubes containing 6 ml saline, and incubated at 37°C for 2 hours to simulate ischemic conditions. The exterior solution for each position was sampled at 0, 30, 60, 90, and 120 minutes and loaded in duplicate (75 µl/well) into a 96 well plate (black-sided flat bottom polystyrene, Corning, New York, NY). Plates were read in a plate reader (FilterMax F-5 Multi-mode, Molecular Devices, Sunnyvale, CA) for concentration of fluorescein (excitation 494/emission 521) to measure diffusion across the wall of the intestine.

After the two-hour ischemic incubation, intestinal pieces from segments 2 (jejunum) and 7 (ileum) were embedded in O.C.T. (Tissue Tek, Torrance, CA) and snap frozen in t-methyl butane in liquid nitrogen. Intestine samples from segments 2 and 7 (for protease analysis) and segment 7 (for immunoblots) were snap frozen and stored for subsequent homogenization.

The small intestines of a separate group of animals were used as *pre-ischemic controls* for morphology and the corresponding regions of tissue were embedded in O.C.T. and frozen. As a non-ischemic control for permeability, a segment of distal ileum (approximating segment 7 which had the greatest permeability in the ex-vivo ischemic case) from a separate group of animals was cannulated, flushed, filled with saline with fluorescent markers, as described above, and immersed in a small saline bath, but the blood supply to the segment was left intact. The animals were maintained under general anesthesia for 2 hours, and the samples of exterior fluid from the saline bath were measured and adjusted for volume dilution in the bath.

#### Permeability Analysis

To determine the fluorescein transport across the ischemic intestinal wall, the fluorescence at time 0 was subtracted from fluorescence values at later time points to correct for background, and fluorescence values were corrected to account for the volume reduction due to sampling during the experiment. The RFU measurements were converted to equivalent moles by using the linear relationship between concentration and fluorescein fluorescence (not shown, R^2^ = 0.999). The transport of fluorescein across the wall (measured in moles, per time) was computed as the change in fluorescence over a 30 min interval divided by 30 min. Individual positions were excluded if the fluorescence measured at time 0 exceeded a pre-selected minimum threshold, suggesting a torn specimen or a specimen with incompletely sealed ends (this occurred 6 times out of a total of 192 intestinal segments).

#### Hemorrhagic Shock Model

Hemorrhagic shock was used to study the ability of treatments, shown in the ex-vivo portion of the study to preserve the barrier and protect the intestine in the absence of luminal content, to prevent intestinal injury after ischemia/reperfusion injury in-vivo when luminal content is present.

Following general anesthesia, the femoral artery and vein were cannulated. Systolic, diastolic, heart rate, and mean arterial pressure (MAP) were recorded throughout the procedure using LabChart (AD Instruments, Dunedin, New Zealand). Rats were randomly divided into the following groups: No-HS (N = 6), HS+Saline (N = 6), HS+Glucose (N = 7), HS+Tranexamic Acid (N = 7), and HS+Tranexamic Acid+Glucose (N = 4). No-HS animals were cannulated and immediately sacrificed for tissue collection.

In the HS groups, anesthetized rats were subjected to laparotomy and the intestine was exposed, and a “*Pre-HS*” image was captured. Using a 5 ml syringe (Becton Dickinson), intraluminal treatments of either saline, 10% glucose in saline, 200 mM tranexamic acid in saline, or the combination of 10% glucose with 200 mM tranexamic acid in saline was warmed to 37°C in a water bath before injecting each treatment in two to three sites along the length of the intestine from the jejunum to the ileum (10.7±1.0 ml; mean±SD). Extra caution was applied to fill the entire length but avoid over-inflation of the intestine. After injection, the intestine was carefully returned to the peritoneal cavity and the wound was covered with moist gauze and plastic wrap to keep the animal warm.

To prevent clotting in catheters and shed blood, animals were heparinized (1 U/ml i.v., assuming 6% blood volume per body weight) prior to inducing hemorrhagic shock. Blood was removed through the femoral venous catheter (0.4 ml/min) until the MAP reached 30 mmHg. MAP was maintained at 30 mmHg by withdrawal/return of blood over the 90-minute ischemia period. At the end of ischemia, the shed blood was returned to the animal (0.5 ml/min). The animal was kept anesthetized and was observed for 3 hours. Gross morphology images of the intestine were captured, “*Post-HS*’.

At the end of reperfusion, segments of intestine (jejunum; 10 cm from ligament of Treitz) were snap frozen for homogenization and embedded in O.C.T. (Tissue Tek).

#### Morphological Analysis and TUNEL Labeling

Intestinal segments frozen in O.C.T. were cut into 5 µm sections. Sections were fixed using ice-cold methanol (8 min at −20°C) and immediately washed four times in distilled water. Nuclei were stained by incubating sections in freshly mixed Weigert's Iron Hematoxylin A and B (Electron Microscopy Science, Hatfield, PA) (10 min) followed by rinsing thoroughly with water. Collagen fibers were labeled by incubating (2 min) in Van Gieson's Solution (Electron Microscopy Science).

To assess the level of apoptosis after ischemia, in-situ terminal transferase dUTP nick end labeling (TUNEL) labeling was completed using a kit (Trevigen, Gaithersburg, MD) according to manufacturer's instructions. Sections were counterstained with 0.05% toluidine blue in 1% boric acid to contrast 3,3′-diaminobenzidine (DAB) positive nuclei (brown) from negatively stained nuclei (blue).

Prior to mounting, all slides were dehydrated (70, 95, 100% ethanol) and cleaned with xylene. After air-drying overnight, sections were mounted using Hard Set Mounting Media (Vector Laboratories, Burlingame, CA). Digital images were captured with a 20× objective (numerical aperture 0.5) and digitally montaged together after background subtraction.

The separation of the mucosal epithelial layer was quantified for groups that had intact villi following the two hour ischemic period. Images were blinded and the distance between the mucosal epithelial layer and the lamina propria was measured over five lengths across a minimum of 6 villi.

### Enzyme Activity

#### Gelatin Gel Zymography

Segments 2 and 7 from pre-ischemic intestines were homogenized (0.1 g tissue/ml homogenization buffer; PBS pH 7.4 with 0.5% hexadecyltrimethyl bromide) and centrifuged at 1.4×10^4^ g for 20 minutes. Supernatants were stored at −80°C until samples were processed.

To assess the proteolytic activities of individual proteases in intestinal tissue samples, sample volumes of 1 µl (with 2 µg of protein) were separated by gel electrophoresis in SDS-PAGE gels containing 80 µg/ml gelatin. Gels were renatured by four 15 min washes with 2.5% Triton X-100 and incubated overnight at 37°C in developing buffer (0.05 M Tris base, 0.2 M NaCl, 4 µM ZnCl_2_, 5 mM CaCl_2_·2H_2_O). After incubation, gels were fixed and stained (50% methanol, 10% acetic acid, 40% water, and 0.25% Coomassie blue solution) for three hours before de-staining in water. The molecular weights of the proteases were estimated by use of a standard protein ladder (Invitrogen). Gels were digitized and bands were analyzed by densitometry in ImageJ (http://rsbweb.nih.gov/ij/).

To determine which of the enzymes present in the intestinal wall are directly inhibited by tranexamic acid, some selected gels were renatured and developed with 20 mM tranexamic acid in their renaturing and developing buffers. These gels were compared to a concurrently run gel processed normally. Additionally, to confirm certain bands inhibited by tranexamic acid were MMPs doxycycline (1 mg/ml) or GM-6001 (1 ug/ml) were added to the renaturing and development buffer for select gels.

#### MPO Activity

Myeloperoxidase (MPO) activity in the intestine as a measure for neutrophil accumulation was selected as an index of reperfusion injury after hemorrhagic shock. Segments from the jejunum (about at the position of segment 2 in the ex-vitro study) were homogenized with their native luminal contents (PBS pH 6.0 in 0.5% HTAB). 40 µl of 2 mg/ml intestine homogenate was added to 180 µl of PBS (pH 6.0) mixed with 0.167 mg/ml o-dianisidine dihydrochloride (Sigma-Aldrich) in duplicate and 0.001% H_2_O_2_ (w/v).

Absorbance was measured kinetically at 450 nm every 5 minutes for 1 hour at 37°C. As negative controls, 180 µl of PBS (pH 6.0) was added to 20 µl of sample and the absorbance values of these samples were subtracted from the measurements with the substrate. The change in absorbance was linear within this period, and MPO activity is presented as the change in absorbance per minute per milligram of protein.

### Immunoblotting

To determine whether selected epithelial barrier proteins were degraded during ischemia, we analyzed pre- and post-ischemic intestinal tissue homogenates (segment 7) for relative levels of mucin 13, occludin, and E-cadherin.

Tissues were homogenized for immunoblot analysis in buffer (CelLytic, Sigma-Aldrich) with protease inhibitor cocktail (HALT, Thermo Scientific, 1∶100 dilution) and centrifuged (1×10^4^ g, 20 min). Protein concentration in supernatants was determined (BCA, ThermoScientific). To separate proteins by gel electrophoresis, 30 µg of protein/well were loaded in 8 or 12% resolving gels with 4% stacking gels and transferred to nitrocellulose membranes (Bio-Rad, Hercules, CA). Membranes were blocked for one hour with either 5% bovine serum albumin (BSA) or 5% nonfat milk in tris-buffered saline with 0.5% Tween-20 (TBS-T). Primary antibodies diluted in 1% BSA or 1% nonfat milk against mucin 13 (1∶1000, extracellular domain, sc-66973 Santa Cruz Biotechnology, Santa Cruz, CA), E-cadherin (1∶1000, intracellular domain, 33-4000, Invitrogen), occludin (1∶1000, intracellular domain, 33–1500, Invitrogen), pancreatic trypsin (1∶1000, sc-137077 Santa Cruz Biotechnology), or β-actin (1∶1000, sc-130301, Santa Cruz Biotechnology) were incubated overnight at 4°C. After incubation, membranes were washed with TBS-T (3×, 10 min) before addition of goat anti-rabbit or rabbit anti-mouse secondary antibodies (Santa Cruz Biotechnology) diluted 1∶1000 in TBS-T. Following secondary antibody incubation (60 min), the membranes were washed with TBS-T (3×) and developed.

### Statistical Analysis

Results in the severe ischemia ex-vivo model are shown as mean ± standard error of mean (SEM) (N = 3–6/group) to display with greater clarity the time courses for the tracer transport. Two factor ANOVA was used to compare between positions (1–8) over time within each treatment group followed by Tukey post hoc analysis to compare each position over time and each position to position 2 (which typically showed the lowest permeability). To compare the saline group to the treatment groups, the data were fit with polynomial equations (third order polynomials gave the best data representation). Inhibitors' equations were compared to saline's equation by the F-test. Single factor ANOVA analysis followed by Tukey post hoc test was used for immunoblot comparisons. Paired t-test was used to compare protease activity in jejunum and ileum segments. Statistical analysis was performed using Origin software (Northampton, MA). Results for hemorrhagic shock are presented as mean±standard deviation (SD).

## Results

### Transmural permeability is attenuated with metabolic support from glucose

**Figure 1:**
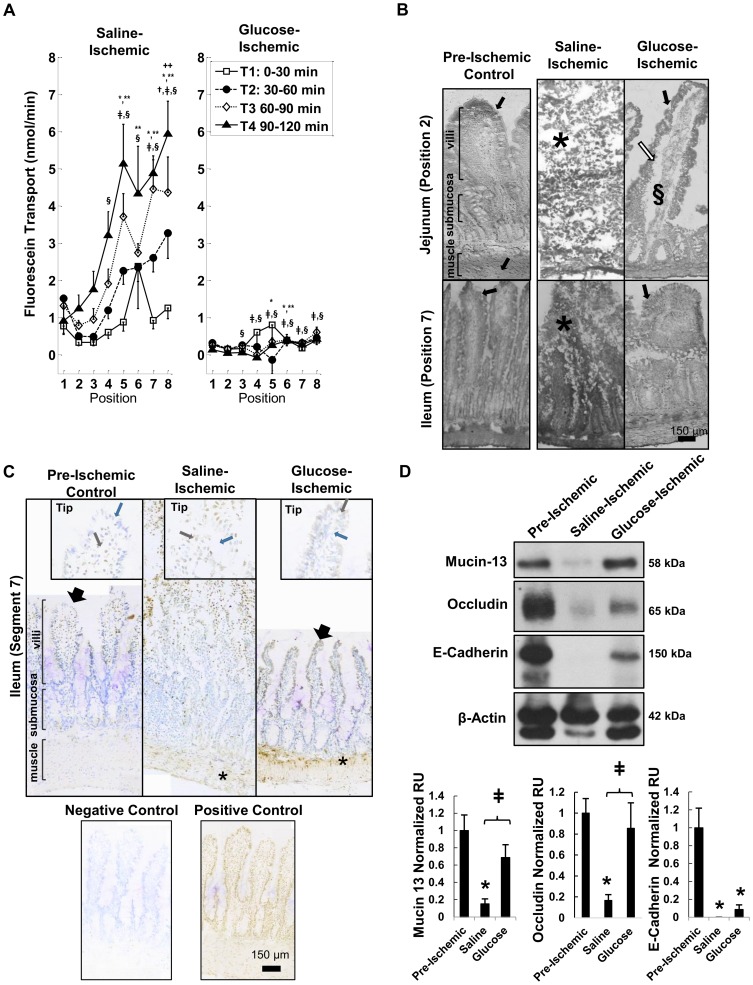
Fluorescein transport and tissue degradation in saline and glucose treated intestines after severe ischemia. The intestinal lumen was flushed of all food contents and digestive enzymes. (A) Fluorescent tracer transport rates across the wall of ischemic intestinal segments located along the length of the small intestine from the proximal jejunum (position 1) to the distal ileum (position 8). The fluorescein transport rates were computed as amount (nM) of fluorescein accumulating outside of the intestinal wall over 30 min time intervals. The intestinal segments were enterally filled with fluorescein in saline without and with glucose before ischemia began. p<0.05 by 2-way ANOVA, with Tukey post hoc test significance compared to Position 2 marked by ++ (during time interval T2), * (T3), ** (T4). p<0.05 by Tukey post hoc test compared to T1 shown by † (T2), ‡ (T3) and § (T4). (B) Intestinal wall morphology in the jejunum (top) and ileum (bottom) as seen on frozen sections after Van Gieson and hematoxylin labeling. Black arrows indicate intact villi structure that best matched the structure of the pre-ischemic tissue, white arrow shows separation between the lamina propria and the mucosal epithelial layer, (*) indicates sites of damaged villi, and (§) specifies internal damage to the villi. (C) TUNEL labeling of pre-ischemic control, saline, and glucose intestine segment and the tips. Black arrows point to intact villi. Brown stained nuclei indicate TUNEL-positive cells (brown arrows) while blue labeled nuclei indicate negative labeling (blue arrows). Positive and negative controls depict brown and blue stained nuclei, respectively. * indicates TUNEL positive cells in the muscularis. (D) Immunoblots of epithelial bound mucin 13, occludin, and E-cadherin. *, p<0.05 vs. pre-ischemic control tissues and ‡ compared to saline tissues by one way ANOVA with Tukey post hoc. N = 6 rats/group for saline and N = 5 rats/group for glucose. Mean±SEM.

During ischemia in the absence of treatment, fluorescein transport across the intestinal wall was positive with increasing fluorescence at all locations along the intestine for every time interval (Figure 1A). The average fluorescein transport from ischemic intestines containing normal saline increased with position along the length of the intestine from the jejunum to the ileum (though position 1 at the proximal jejunum generally had a higher transport than position 2) (Figure 1A). Glucose administration into the lumen of the intestine drastically reduced the fluorescein transport across the wall of the intestine even after 2 hours (Figure 1A). Ischemic conditions in the saline group led to destruction of the intestinal villi structure both in the jejunum and the ileum (Figure 1B). This was attenuated in the glucose group; however, we still observed separation between the lamina propria and the mucosal epithelial layer and *internal* damage to the villi.

Pre-ischemic control intestines had little apoptosis except near the tips of the villi (Figure 1C) while saline-treated ischemic intestines showed extensive apoptosis in cells in the villi as well as cells detached from the villi (nuclei are visible despite loss of villi structure). Intestines treated with glucose in the lumen had little apoptosis in the villi, though TUNEL labeling similar to that in the saline group was present in the muscle layer (Figure 1C). After severe intestinal ischemia, mucin 13 bound to the epithelium and occludin decreased in the saline group but were preserved in the presence of luminal glucose (Figure 1D). E-cadherin decreased irrespective of the presence of glucose (Figure 1D), suggesting it is not involved in the preservation of the epithelial barrier to fluorescein during severe ischemia.

To confirm the beneficial effects of glucose were not a consequence of increased osmolality, mannitol, a non-metabolizable sugar, was substituted for glucose. Mannitol neither prevented fluorescein penetration nor preserved villi structure (not shown), in line with previous findings [33], [39].

When the fluorescein transport rate was measured in-vivo in an isolated non-ischemic ileum (segment 7) with intact perfusion, a small reverse convective flux of fluid from the saline bath into the vasculature and lymphatics of the intestine was detected (resulting in fluorescein *influx* of 0.05±0.06 nM/min at the 90–120 min time point, N = 3 rats/group). In contrast, in the ischemic intestine (also in segment 7) only efflux of fluorescein was seen in the ex-vivo saline group (4.83±0.36 nM/min, N = 6 rats/group). The villi in the vascularized control were intact at the two-hour collection time point (not shown).

### Protease activities of intestinal homogenates

**Figure 2:**
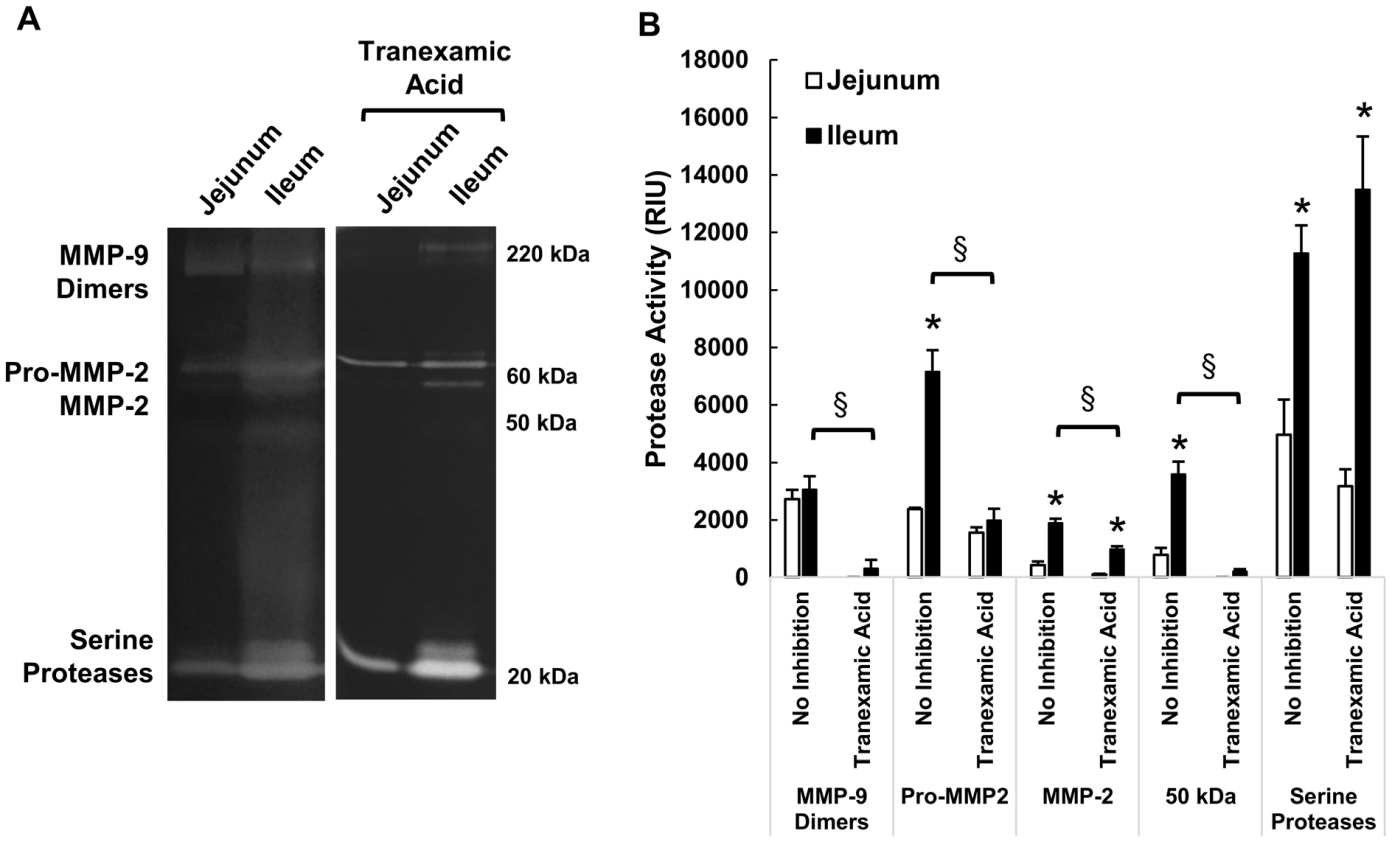
Protease activity in intestinal homogenates. (A) Gelatin gel zymographies showing protease activity in the pre-ischemic jejunum (segment 2) and ileum (segment 7), with and without renaturation in tranexamic acid. (B) Quantification of bands by densitometry. *, p<0.01 by paired t-test between jejunum vs. ileum. §, p<0.01 by paired t-test between No Inhibition and Tranexamic Acid (20 mM) renaturing. N = 4/group. Mean±SEM.

The proteolytic activity profile between the jejunum (segment 2) and ileum (segment 7) differed significantly (Figure 2A) as determined by gelatin gel zymography in pre-ischemic control tissue homogenates (Figure 2A). Many of these bands were reduced by addition of tranexamic acid to the renaturing and developing solutions (Figure 2A). Though reported primarily as a plasmin inhibitor [40], tranexamic acid was nevertheless able to significantly reduce the bands that formed in the ileum around 220 kDa corresponding to MMP-9 dimers, pro-MMP-2, MMP-2, and the 50 kDa band corresponding to either MMP-1 or MMP-3 [23], but had no significant effect on the 20 kDa serine proteases, which likely contain more serine proteases (e.g. chymotrypsin) than just the trypsin-like proteases expected to be inhibited by tranexamic acid (Figure 2B). Protease activity bands still formed with equal intensity in the ileum even if only the ileum were flushed indicating that the increased activity in the ileum is not arising from the passage of jejunal contents (not shown). As positive controls, gels were renatured with either doxycycline or GM-6001 and MMP bands were reduced or eliminated and serine protease bands remained in all gels for all samples (data not shown).

Intestine tissue homogenates from the 2 hr ischemic saline treated group had no change in the protease activity profile by gelatin gel zymography compared to the pre-ischemic intestinal homogenates (results not shown).

### MMP inhibition reduces transmural permeability

**Figure 3:**
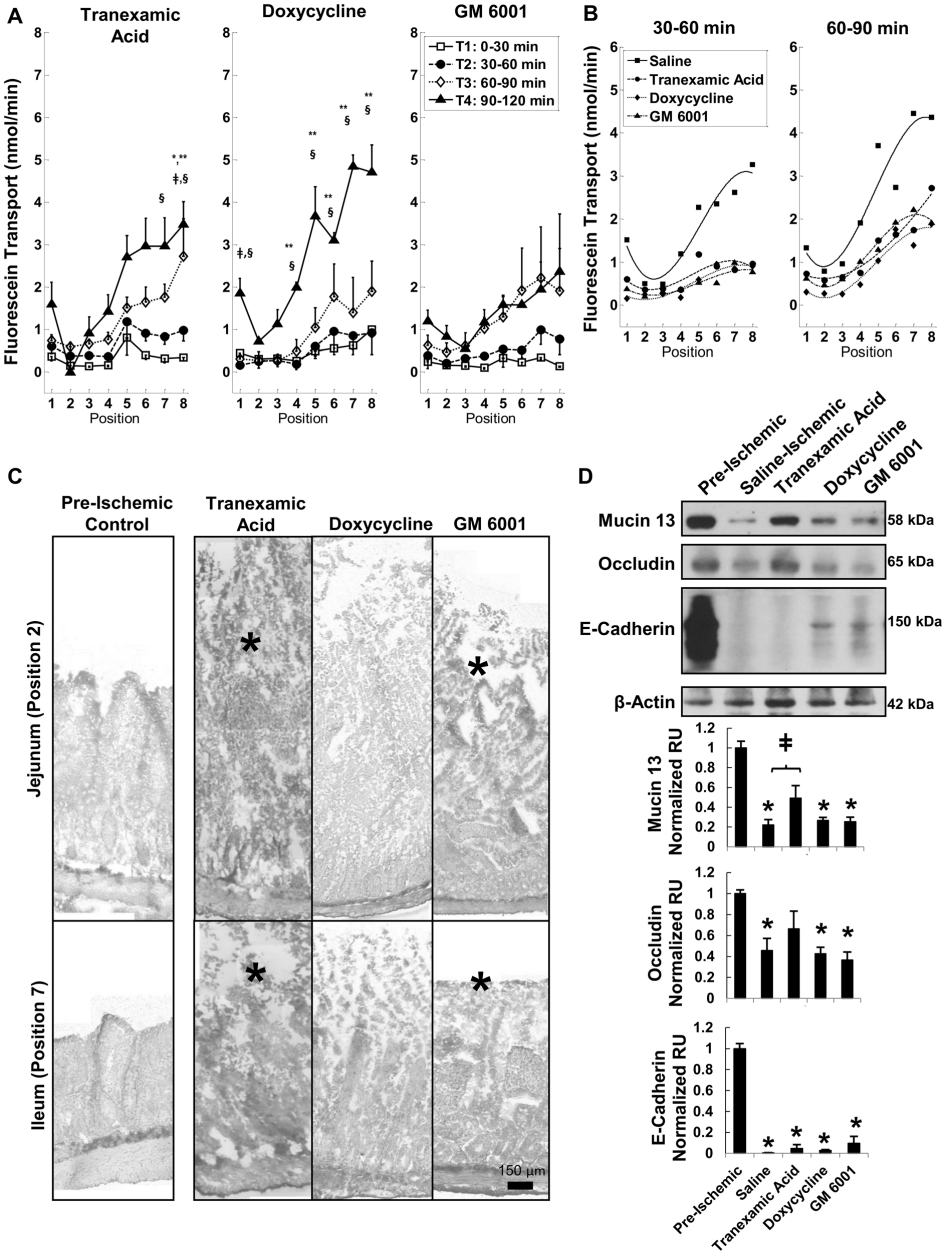
MMP inhibition and intestinal wall destruction during ischemia. (A) Fluorescein transport across the wall of ischemic intestinal segments filled with tranexamic acid, or MMP inhibitors (doxycycline, GM 6001). p<0.05 by two way ANOVA with Tukey post hoc test significance compared to Position 2 shown by ** (during T4). Significant changes (p<0.05 by Tukey post hoc test) compared to T1 shown by ‡ (T3) and § (T4). N = 3 rats/group. (B) Third order polynomial fit to measured mean fluorescein rates at each position for either the 30–60 min or 60–90 min period for tranexamic acid, doxycycline, GM 6001 and saline groups to compare fluorescein transport at 30–60 and 60–90 min of severe ischemia. Adjusted R^2^ values are 0.87, 0.37, 0.81, and 0.71 for 30–60 min; 0.80, 0.89, 0.97, and 0.86 for 60–90 min for saline, tranexamic acid, doxycycline and GM 6001 curves, respectively. Comparison of curve fits for tranexamic acid, doxycycline or GM 6001 with those for saline by F-test, p = 5.6×10^−4^, 1.3×10^−4^ and 9.1×10^−5^ for 30–60 min; p = 6.5×10^−3^, 1.8×10^−3^ and 4.3×10^−3^ for 60–90 min. (C) Representative images of the intestinal villi. Arrows indicate intact villi structure and (*) indicates sites of damaged villi after ischemia. (D) Immunoblots of epithelial bound mucin 13, occludin, and E-cadherin. *p<0.05 vs. pre-ischemic control tissues and ‡ compared to saline-ischemic tissues by one way ANOVA with Tukey post hoc. N = 6 rats/group for pre-ischemic controls and tranexamic acid; N = 3 rats/group for doxycycline and GM-6001. Mean±SEM.

Inhibition with tranexamic acid or the broad-spectrum MMP inhibitors doxycycline and GM 6001 prevented significant increases in fluorescein transport at early, but not later time points (Figure 3A). The transport rates in the doxycycline and GM 6001 group closely matched the tranexamic acid group in the 30–60 min and 60–90 min periods and were significantly decreased compared to the saline treated intestines (Figure 3B). However, in the 90–120 min period, GM 6001 was more effective at reducing the transmural permeability compared to either doxycycline or tranexamic acid (not shown). The villi in the jejunum and ileum were not intact after 2 hrs of ischemia (Figure 3C).

In the ex-vivo model, neither doxycycline nor GM 6001 were effective at preserving mucin 13, occludin, or E-cadherin breakdown at the two hour time point, but tranexamic acid was able to preserve mucin 13 levels and prevent a significant decrease in occludin (Figure 3D), even though the cells were no longer attached to intact villi.. This suggests that MMPs are not directly involved in the breakdown of the mucosal barrier during severe ischemia (i.e. their early benefits to transmural permeability could reflect preservation of *other* barriers to diffusion such as ECM proteins or cell/matrix or cell/cell adhesions in the muscle layer). In contrast, tranexamic acid may provide some additional benefit directly to the mucosal barrier, especially if it were combined with glucose. Since mucin plays a role in protection of the intestine when luminal content is present, tranexamic acid in this model may be a better choice for intestinal protection in-vivo than an inhibitor that targets MMPs alone.

### Hemorrhagic shock with enteral glucose or tranexamic acid

Following this ex-vivo analysis, we applied the interventions most effective at minimizing intestinal destruction in the clinically more relevant model of hemorrhagic shock with an *unflushed* intestine. Tranexamic acid was chosen over GM 6001 because it was able to preserve mucin 13, reduce the separation of the mucosal epithelial layer from the lamina propria, and does not require solubilization in an organic solvent (DMSO).

**Figure 4:**
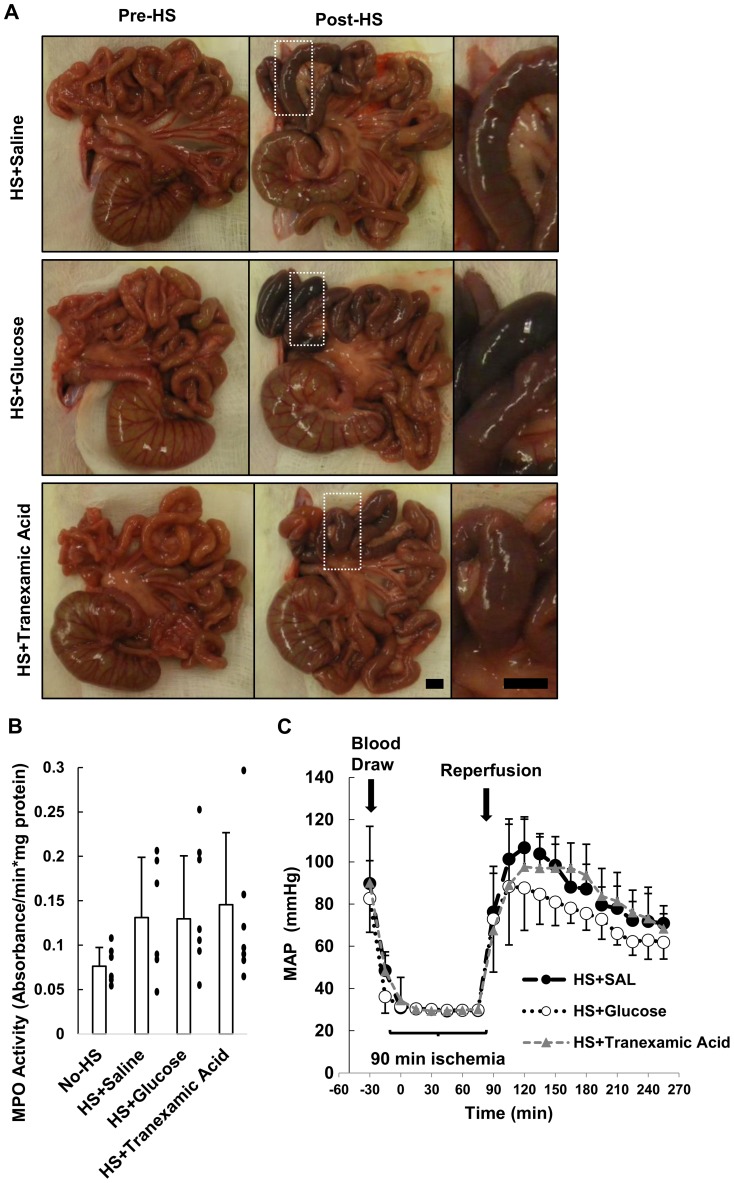
Glucose or tranexamic acid intervention in hemorrhagic shock. (A) Gross morphology of the intestine in the rats before and after hemorrhagic shock. Lesions due to escape of red cells form in the HS+Saline animals and HS+Glucose treated animals (see magnified views) while the HS+Tranexamic Acid animals intestinal injury was in part reduced. (B) MPO activity measured in intestinal segments from segment 2, the region with the appearance of the most severe lesions, was elevated in all groups after hemorrhagic shock. “•” indicate individual data points for each animal. (C) Mean arterial pressure (MAP) of animals during the course of hemorrhagic shock. Data are presented as mean±SD. N = 6 rats/group for No-HS and HS+Saline; N = 7 rats/group for HS+Glucose and HS+Tranexamic Acid. Scale bar equals 5 mm. Mean±SD.

For the first part of this study, we pre-treated the lumen of the intestine before the onset of hemorrhagic shock with single treatments only. After hemorrhagic shock, all animals regardless of pretreatment with saline, glucose, or tranexamic acid had lesions form in the jejunum (Figure 4A). The neutrophil accumulation in the jejunum was elevated after HS in all groups (Figure 4B). The MAP decreased linearly during 2 hours of reperfusion in all cases after HS at rates of −0.32±0.12 mmHg/min, −0.21±0.13 mmHg/min, −0.27±0.16 mmHg/min for HS+Saline, HS+Glucose, and HS+Tranexamic Acid, respectively. The glucose group had on average a lower starting MAP and final MAP due to the parasympathetic activity of glucose [41], [42].

Histological sections of the intestine after hemorrhagic shock showed no destruction of the villi, unlike the severe ischemic model. There was no mucin 13 or occludin degradation after shock in intestinal homogenates confirming the inherent differences between the ex-vivo model and the hemorrhagic shock models (data not shown). We next examined the effect of combining the metabolic support with protease inhibition treatment in both the ex-vivo severe ischemia and hemorrhagic shock models.

### Gut protection with tranexamic acid+glucose after hemorrhagic shock

**Figure 5:**
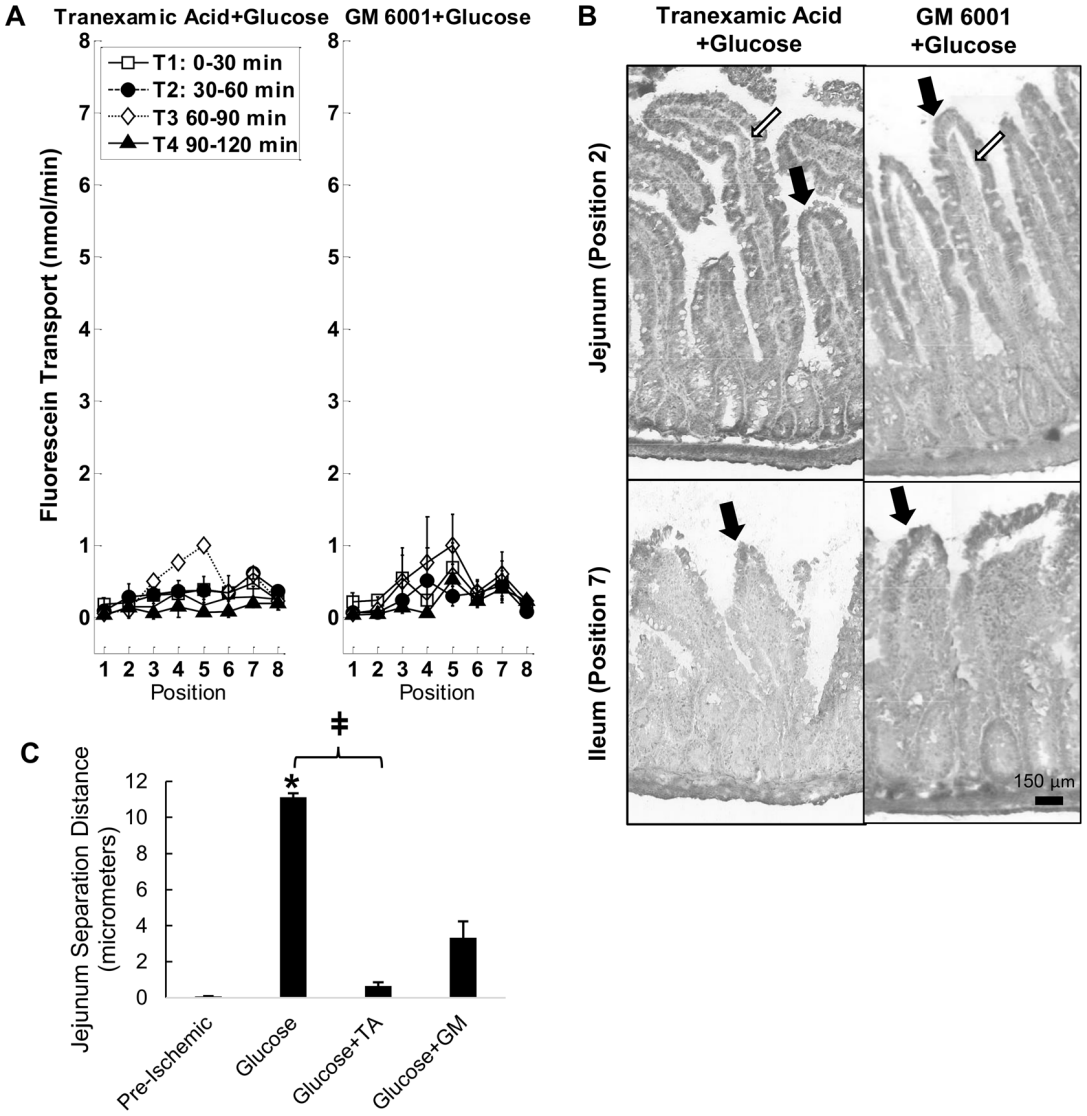
Tranexamic acid or GM 6001 combined with glucose fluorescein transport. (A) Fluorescent tracer rates across the wall of ischemic intestinal segments filled with tranexamic acid+glucose or GM 6001+glucose in saline. N = 3 rats/group. (B) Representative micrographs of intestinal villi with tranexamic acid+glucose or GM 6001+glucose after ischemia. Black arrows indicate intact villi structure similar to the pre-ischemic control (Figure 1) and white arrows indicate points of separation between the lamina propria and the mucosal epithelial layer. (C) Separation between the lamina propria and the mucosal epithelial layer. *, p<0.0001 vs. pre-ischemic intestinal tissue. Refer to Figure 1B for images of pre-ischemic and glucose treated intestines. ‡, p<0.0001 for glucose vs. glucose+tranexamic acid. N = 3 rats/group. Mean±SEM.

The combination of tranexamic acid+glucose and GM 6001+glucose (Figure 5A) served to maintain low permeability throughout the two-hour ischemic period and did not behave differently than glucose alone. Because of the glucose, the villi in the intestine also remained intact following 2 hours of intestinal ischemia (Figure 5B). However, unlike glucose alone, the epithelial layer stayed adhered to the lamina propria in the jejunum significantly better when tranexamic was combined with glucose (Figure 5C). GM 6001+glucose resembled the glucose case but did not protect against the separation from the lamina propria as effectively.

**Figure 6:**
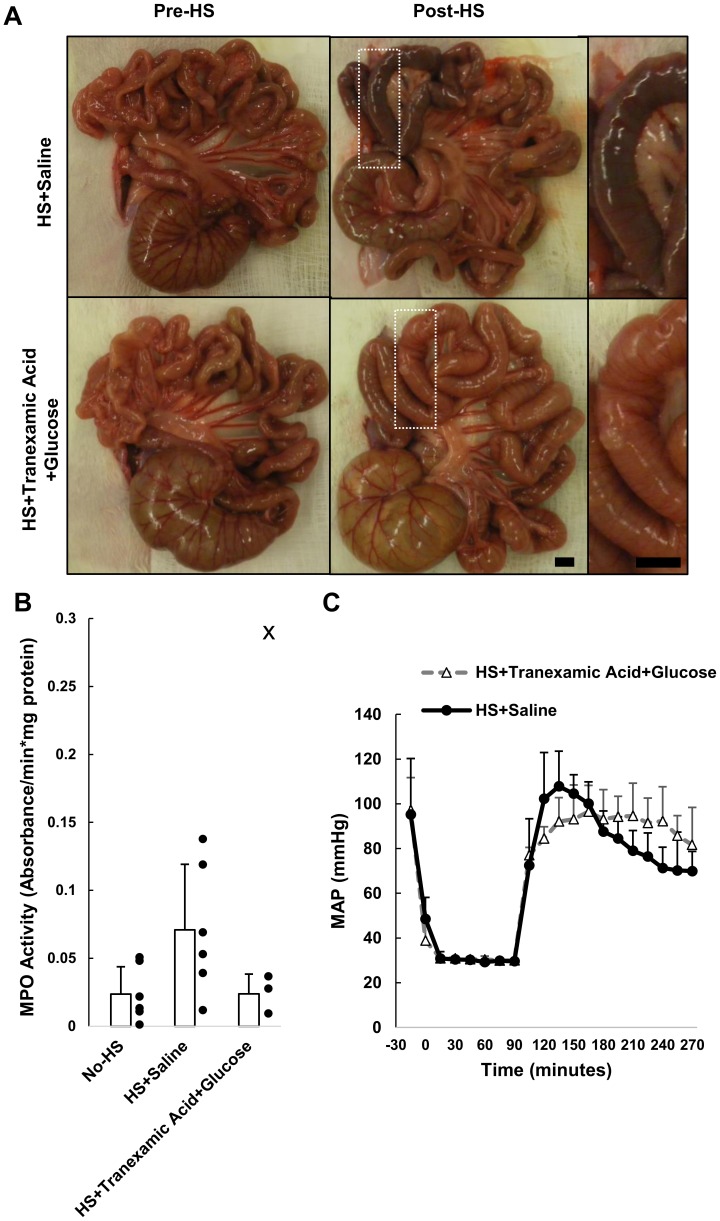
Hemorrhagic shock with enteral tranexamic acid+glucose. (A) Gross morphology of the intestine in rats before and after hemorrhagic shock. Lesions form in the HS+Saline animals but were reduced in the HS+Tranexamic Acid+Glucose treated animals (see magnified views). (B) MPO activity measured in intestinal segments from segment 2 trended to decrease in the HS+Tranexamic Acid+Glucose animals. “•” indicate individual data points for each animal and ‘x’ indicates an outlier. Bar graph shows mean±SD; outlier is excluded from the bar graph mean value in the HS+Tranexamic Acid+Glucose group. (C) Mean arterial pressure (MAP) of animals during the course of hemorrhagic shock. The MAP during the reperfusion period followed a linear trend for the HS+Saline animals. N = 6 rats/group for HS+Saline; N = 4 rats/group for HS+Tranexamic Acid+Glucose. Scale bar equals 5 mm. Mean±SD.

Since the dual treatments appeared to maintain an epithelial layer and prevent internal tissue degradation, we tested the combination of glucose and tranexamic acid in hemorrhagic shock. The intestine appeared healthy in all animals prior to the onset of shock with no evidence for hemorrhage or distensions (Figure 6A). The majority of the lesions in the jejunum region were reduced if tranexamic acid+glucose were administered simultaneously rather than independently (Figure 4A and Figure 6A).

We sought to measure the degree of neutrophil infiltration into the intestine after shock and found that the animals treated with tranexamic acid and glucose did not have significantly lower MPO activities, though there was one outlier with high MPO activity (Figure 6B). The animal with the greatest MPO activity in the HS+Tranexamic Acid+Glucose (outlier) also had macroscopic hemorrhages similar to the HS+Saline animals.

The MAP in the reperfusion phase shows that the HS+Saline animals had a higher initial pressure at the start of reperfusion that decreased at a faster rate compared to the HS+Tranexamic Acid+Glucose treated animals (Figure 6C). Comparing the average regression between the groups of the MAP drop from the last 2 hours of reperfusion, HS+Saline animals decreased at a rate of −0.32±0.12 mmHg/min (mean±SD), which was significantly faster (p<0.005 by t-test) than the HS+Tranexamic Acid+Glucose animals which decreased at a slower rate of −0.07±0.08 mmHg/min (mean±SD).

## Discussion

### Summary

The current results indicate that the transmural transport of a low molecular weight tracer from the lumen across the rat intestinal wall was undetectable in a non-ischemic state but increased after complete intestinal ischemia, even in the absence of its luminal contents. The permeability increased consistently from the jejunum to the ileum in all cases and may be a result of the increased basal protease activity in the ileal tissue (Figure 2). Placement of glucose into the lumen of the small intestine abrogated the transmural permeability increase of a low molecular weight tracer, reduced apoptosis and loss of the tight junction protein occludin, and served to maintain the epithelial layer but not necessarily the underlying intestinal wall (Figure 1B, also reported by Derikx, et al.) [43]. In the absence of glucose, the MMP inhibitors doxycycline and GM 6001 and the protease inhibitor tranexamic acid reduced the transmural permeability through the 60–90 min time interval, but they did not prevent the villi destruction at 2 hrs. In acute hemorrhagic shock, unflushed intestines enterally pre-treated with tranexamic acid+glucose had reduced formation of macroscopic lesions and stabilized blood pressure compared to animals with enteral saline only. The evidence suggests that both the epithelium and the tissue under the epithelium act as barriers to the transmural passage of a low molecular weight tracer, and preventing both avenues of damage is necessary to reduce the intestinal injury after hemorrhagic shock.

### Glucose preserves the epithelial barrier by preventing epithelial cell shedding

Glucose administration curtailed the rise in transmural permeability to fluorescein in the ischemic small intestine, likely by maintaining the epithelial barrier, in agreement with previous results showing preservation of the barrier properties during ischemia in the presence of luminal glucose [31]–[33]. Despite blood flow cessation, glucose provides epithelial cells a direct source of metabolic energy to produce ATP [30], which has been shown to maintain the barrier in-vitro [44]. ATP supply, which could be achieved by glucose supplementation, is required for epithelial cells to maintain attachments and prevent excessive apoptosis [32], [45]–[47] and shedding of apoptotic epithelial cells into the lumen (anoikis) [5]. The presence of glucose in the lumen during ischemia prevented the cells from undergoing apoptosis (Figure 1C). Elevated levels of apoptosis and epithelial shedding into the lumen can occur within 15 minutes of intestinal ischemia [5], which illustrates the sensitivity to ischemic conditions.

### Partial protection with MMP inhibition may be due to preservation of extracellular matrix

The intestine is rich in MMPs, and we detected gelatinase activities at approximately 50 kDa (MMP-1 or MMP-3), 60 kDa (MMP-2), and 220 kDa (MMP-9 dimer) similar to previous findings [23], [50]. MMPs could originate from a variety of cells in the intestine, including immune cells such as neutrophils and mast cells in the intestine [23], [50], [51]. Doxycycline and GM 6001 are both broad-spectrum MMP inhibitors [52] (GM 6001 also inhibits ADAMs [38]). Unexpectedly, tranexamic acid was also able to directly inhibit MMPs (Figure 2). All three inhibitors reduced but did not prevent some increase in transmural permeability through the first 90 minutes of ischemia. By 120 minutes, permeability was no longer significantly lower than that in the saline group. It is currently unclear how much of this early preservation was due to protection of the intestinal extracellular matrix versus reduced destruction of the epithelial layer (Figure 2B), since both the extracellular matrix and epithelial layer were damaged after 120 min of ischemia ex-vivo. However, given that protection of the epithelial layer by glucose completely prevented permeability increase, it is likely that the epithelial barrier is the dominant barrier that controls transmural permeability and that partial prevention of permeability increase with MMP inhibition is due instead to effects on the extracellular matrix. Protection of extracellular matrix proteins from degradation may minimize the creation of pores through the lamina propria, muscularis, and serosa that fluorescein could use to reach the outer compartment [22], even if the epithelial barrier were no longer intact.

In support of this idea, the tight junctional protein occludin was decreased by ischemia and preserved by glucose, but not doxycycline or GM 6001. Interestingly, E-cadherin, which was also decreased by ischemia, was not preserved with glucose, supporting that epithelial barrier properties are maintained by tight junctions rather than by adheren junctions. Occludin and E-cadherin can be degraded by MMPs [53]–[55]; however, MMP inhibition did not curtail occludin or E-cadherin destruction, implying MMPs are not responsible for their reduction following ischemia. Alternatively, as the epithelium becomes apoptotic, occludin, E-cadherin, and mucin 13 may be internalized and subsequently degraded [18], [56], possibly aided by lactic acid build up and reduced intracellular pH during ischemia [57]. It should be noted that E-cadherin and mucin 13 also degrade in the presence of luminal proteases [6], [18], and therefore, their breakdown could be accelerated in cases of severe ischemia when luminal content is also present [11].

Tranexamic acid had additional protective effects that were not present with the other MMP inhibitors. It preserved mucin 13, which could improve epithelial survival in non-flushed intestines [6], [18], and the decrease in occludin levels, while not significantly better than saline animals, was also not significantly different from pre-ischemic levels. Histology from the combined treatment of tranexamic acid with glucose suggests that tranexamic acid may also help the preservation of epithelial attachments to the basement membrane in the ex vivo study (Figure 5C), which may reduce anoikis, helping to preserve the epithelial barrier component. Reduced anoikis could therefore explain the improvements in mucin 13 and occludin. Likely, these additional benefits over doxycycline and GM 6001 are due to the fact that tranexamic acid, as a lysine analog, inhibits plasmin and conversion of plasminogen to plasmin. Plasmin is a trypsin-like enzyme that is a potent activator of MMPs [48], [49] and ADAMs (“a disintegrin and metalloproteinase”) expressed on epithelial cells and activated during ischemia [49]. Therefore, inhibiting plasmin may also attenuate the consequent downstream activation of metallo- or other proteases that contribute to tissue destruction during ischemia.

### Jejunum and ileum have different permeability profiles

In all cases, the transmural permeability for fluorescein increased from the jejunum to the ileum (with a non-significant increased permeability in position 1 compared to position 2). The major transition from an impermeable to a permeable state occurred after 1 hour in positions 5 and higher, corresponding to the ileum. The ileum, often recognized as the most permeable region of the intestine [29], [35], is associated with the most damage after trauma and/or shock and is where the majority of microhemorrhages in experimental shock animals and necrotizing enterocolitis occur [58]–[61]. We were surprised that the morphology of the untreated ischemic jejunum appeared as disrupted as that of the untreated ischemic ileum, despite its lower transmural permeability. Since the epithelial layer, which serves as the primary barrier to fluorescein, was destroyed in both cases, the differences in tracer permeation between the jejunum and ileum lie beneath the epithelium.

When we measured the proteolytic activity by gelatin gel zymography, the wall of the ileum had a higher density of serine proteases and MMPs compared to the jejunum in pre-ischemic tissue (Figure 2). These proteases could degrade the extracellular matrix structure of the villi, muscularis, and/or serosa more rapidly in the ileum than in the jejunum allowing fluorescein to pass with less resistance. Given that the difference between ileum and jejunum was primarily in magnitude of activity rather than composition of proteases, it is likely that if the ischemic period were extended, the jejunum would reach the same high levels of permeability as the ileum. The enhanced presence of proteases in the ileum could cleave integrin attachments between cells and the extracellular matrix, which may enhance apoptosis in the wall of the ileum at a greater rate than the jejunum [62]. Detachment of cells from the extracellular matrix would provide an alternative route for fluorescein to penetrate through the intestinal tissue. Although the transmural permeability of the jejunum is lower than the ileum, it would not necessarily prevent egress through the blood or lymph in-vivo.

### Application of tranexamic acid+glucose to preserve the ischemic intestine after hemorrhagic shock

Our ex-vivo model was designed with a *flushed* intestine with no reperfusion. For the in vivo study we wanted to evaluate the treatment groups in an environment with normal luminal content to more closely match actual trauma situations [11]. We therefore tested the efficacy of saline, glucose, tranexamic or the combination of the two in the lumen of the intestine as a pretreatment for hemorrhagic shock in an *unflushed* intestine. Single interventions of either glucose or tranexamic acid did not prevent microhemorrhages from forming in the jejunum, decrease the neutrophil accumulation in the intestinal homogenates, or stabilize the MAP compared to saline alone (Figure 4) which suggest that even though individual components of the gut may be preserved, the gut reperfusion injury can still occur.

While combined tranexamic acid and glucose treatment improved the overall gross morphology of the intestine (Figure 6A), lowered neutrophil infiltration in three of the four animals (Figure 6B), and stabilized blood pressure (Figure 6C), the presence of an outlier suggests there are other factors in the intestine that can attract neutrophils following ischemia/reperfusion injury. One such factor could be the presence of food, since these animals were eating ad libitum prior to the experiment. We have seen previously that a greater generation of pro-inflammatory mediators in the gut may occur in regions containing partially digested food [17]. Therefore the contents of the intestine may be important in the treatment and diagnosis of patients entering the intensive care unit after traumatic situations. Although tranexamic acid+glucose did reduce intestinal damage, future work needs to determine what components in the lumen attract neutrophils and how these components cause inflammation even in the presence of interventions that reduce transmural permeability and intestinal breakdown.

The transition from reversible to irreversible shock may be related to the severity of the epithelial barrier destruction and the extent to which full physiological gut barrier/digestive features can be restored [63]. Therefore, patients undergoing elective surgery that anticipate intestinal ischemia may benefit from intestinal protection with intraluminal glucose supplementation in combination with tranexamic acid treatment in order to reduce intestinal damage [17], [31], [64].

### Limitations

Flushing the lumen was an effective tool to uncover factors that are responsible for degradation of the wall of the intestine. However, when there are luminal proteases present, the breakdown process may be occurring by multiple protease systems in the lumen and in the wall of the intestine which warrants future investigations.

### Conclusions

The increase in transmural permeability to small molecules during total ischemia of the small intestine occurs by a combination of epithelial shedding and MMP-derived proteolysis of the intestinal wall, both of which occur in the absence of luminal contents (e.g. food, digestive enzymes, bacteria). The rise in transmural permeability during ischemia was reduced by placement of glucose into the lumen of the intestine, as well as by administration of tranexamic acid or MMP inhibitors. Dual treatment preserved intestinal histology (both the overall villi structure and the connection between epithelium and lamina propria) after complete ischemia. Dual treatment, but not individual treatments, with glucose and tranexamic acid in a model of hemorrhagic shock prevented visible intestinal hemorrhage and stabilized blood pressure during reperfusion. Our results suggest that prophylactic treatment with enteral glucose and/or MMP inhibition in trauma patients at high risk for intestinal ischemia may reduce intestinal permeability and transmural passage of luminal content to improve the outcome after ischemic injury.
